# Protective Effect of Breastfeeding against Overweight Can Be Detected as Early as the Second Year of Life: A Study of Children from One of the Most Socially-deprived Areas of Brazil

**Published:** 2015-03

**Authors:** Monica L. Assunção, Haroldo S. Ferreira, Sônia B. Coutinho, Leonor M.P. Santos, Bernardo L. Horta

**Affiliations:** ^1^Faculty of Nutrition, Federal University of Alagoas. Maceió, Alagoas, Brazil; ^2^Postgraduate Programme in Nutrition, Faculty of Nutrition, Federal University of Alagoas. Maceió, Alagoas, Brazil; ^3^Postgraduate Programme in Child and Adolescent Health, Federal University of Pernambuco, Recife, Pernambuco, Brazil; ^4^Department of Public Health, University of Brasília, Brasília, Federal District, Brazil; ^5^Postgraduate Programme in Epidemiology, Federal University of Pelotas, Pelotas, Rio Grande do Sul, Brazil

**Keywords:** Bioactive factors, Childhood overweight, Children, Brazil

## Abstract

Millions of children live in Brazil's semi-arid region, one of the most socially-deprived areas of the country, where undernutrition co-exists with obesity as a consequence of the nutrition transition. There is evidence that childhood obesity predisposes adult obesity and, thus, that obesity should be prevented as early as possible. Some studies have shown that breastfeeding is a protective factor against overweight and obesity while other studies have not found this association. There have been few studies on this association in developing countries and of children below two years of age. The present study aimed to investigate whether children exposed to exclusive breastfeeding for ≥6 months showed a lower prevalence of overweight in the second year of life, based on a probability sample of 2,209 children (aged 12 to 24 months). The dependent variable was overweight, defined as weight-for-length z-scores of >2, based on the WHO 2006 standard while the independent variable was exclusive breastfeeding (≥6 months). The prevalence ratio (PR) and its 95% CI were estimated using Poisson regression with robust adjustment of variance. After adjusting for potential confounding factors (socioeconomic, demographic and health-related variables), children on exclusive breastfeeding for ≥6 months showed a lower prevalence of overweight (5.7% vs 9.1%, PR 0.62, 95% CI 0.45-0.89). It was found that exclusive breastfeeding for six months or more is a protective factor against overweight in children in the second year of life living in the Brazilian semi-arid region.

## INTRODUCTION

Several studies have reported that overweight children are more likely to become obese adults (1,2), reinforcing the importance of early obesity prevention. The challenging question is how and when to intervene to prevent obesity in childhood (3). The rapid increase in the early prevalence of excess weight and its potential effects on morbidity and mortality during childhood and adult life underscores the importance of identifying critical periods for the prevention of excess weight in vulnerable populations.

Regarding how to prevent childhood overweight, the potentially-protective effect of breastfeeding could make it a valuable strategy (4,5). However, while many studies have reported that breastfeeding decreases the risk of obesity (6,7), other studies have not found this to be the case (8–11). Childhood overweight continues to be a pressing issue, not least because an increasing prevalence of obesity has also been reported in developing countries where few studies have been conducted to investigate this relationship (12).

The Brazilian semi-arid region covers an area of 1,142,000 sq km, made up of about 1,500 municipalities in 11 states with an estimated population of 26.4 million. According to UNICEF (13), 81% of Brazilian *Quilombola* communities (descendants of fugitive African slaves) live in this region. Infant mortality is higher than the national average in 95% of the municipalities in this region and perinatal conditions, acute respiratory infections, and nutritional deficiencies account for 33.8% of deaths in children below one year of age. The population of this region is undergoing a nutrition transition, resulting in a reduced prevalence of stunting, alongside rising rates of obesity (14). However, it seems that the transgenerational effect of stunting (15) is still affecting child growth and development.

The present study aimed to investigate whether exclusive breastfeeding for ≥6 months had a protective effect against overweight in children aged 12 to 24 months living in the most socially-deprived semi-arid region of Brazil.

## MATERIALS AND METHODS

### Study design

The study was based on secondary data from the “Health and Nutrition Day” survey which evaluated children living in the semi-arid region of Brazil. The methods have been detailed elsewhere (16).

### Sample

A multistage sampling approach was employed. Each state was a separate domain (N=9), and 30 municipalities were selected per state. In each municipality surveyed, two vaccination centres were randomly selected as secondary sampling units. At each centre, children queuing for vaccination were systematically selected. This method produced a strict probability sample of 16,934 randomly-selected children. The subsample of 2,581 children aged 12 to 24 months was the group of interest for this study. Information on breastfeeding duration was missing for 372 of the children (14.4%), reducing the studied population to 2,209 children in the second year of life.

### Data collection

Data collection took place during National Immunization Day on 20 August 2005. It was conducted by local health professionals recruited and trained especially for the survey. After immunization, the children underwent an anthropometric examination and their parents/caregivers were interviewed. Information on demographic, socioeconomic, anthropometric and health-related variables was collected by the interviewers, using a pretested questionnaire.

Recumbent length was assessed using wooden CARCI infantometers with a measuring range of 10 to 99 cm and graduations of 1 mm, and weight was assessed using a scale with 150 kg capacity and 100 g precision. Measurements of weight and length were taken twice for each child by two trained and supervised examiners.

The sets of questionnaire were coded, and 30% of these were double-checked by supervisors. These were then scanned, and the data were entered into a database. Range and consistency checks were carried out during the coding stage and after data-entry.

### Study variables

Body-weight, length, age, and sex were combined to build anthropometric indices, expressed as z-scores, using Anthro 3.2.2 software package, which includes the WHO 2006 anthropometric references (17). Stunting was defined as length-for-age z-scores of <−2. Overweight was defined as weight-for-length z-scores of >2 (18). The occurrence of overweight due to malnutrition with oedema in Brazil was highly unlikely in 2005; in the 1960s, kwashiorkor was reported in the Northeast but this condition became very rare from 1970 onwards (19). Even so, the health personnel involved in data collection were trained to detect such cases.

Birthweight was categorized as low (<2,500 g), underweight (2,500-2,999 g), normal (3,000-3,999 g), and high (4,000 g or more) (20).

Socioeconomic conditions were evaluated using the Brazilian Economic Classification Criteria (21). Using a series of 10 questions on the household items owned by the respondents (radio, television, refrigerator, vacuum cleaner, washing machine, etc.) and the level of education of the head of the family, a score was calculated, and the respondents were placed in one of the five economic levels, from A (highest) to E (lowest).

Exclusively-breastfed (exc-BF) children were those who received only breastmilk but no water, tea, juice, or any other type of milk or food (22).

### Data analyses

In the analysis, the dependent variable was overweight, and the independent variable was exclusive breastfeeding (categorized as ≥6 months or <6 months).

Means and standard deviations were calculated for the weight-for-length z-scores according to the breastfeeding categories. Levene's test was applied to verify the homogeneity of variances and when there was no significant difference (p>0.05). Student's *t*-test for independent samples was used for comparing means between groups.

In the crude analysis, we tested the association between overweight, exc-BF, and covariates (socioeconomic, demographic and health-related variables) based on the prevalence ratio (PR) and 95% confidence intervals (95% CI). In order to control for potential confounding factors, the variables with p<0.2 in the crude analysis were included in the Poisson regression model with robust adjustment of variance to calculate adjusted PR and 95% CI (23). These variables were: socioeconomic classification (Class A + B + C; D; E); skin colour/race (white/non-white); age (in months; continuous variable); height-for-age (in z-score; continuous variable); birthweight (low/underweight/normal/high weight); and prenatal care (yes/no).

Multiple linear regressions were used for assessing the effect of exc-BF on weight-for-length z-scores, controlling for potential confounding factors. For adjustment, the same covariates as included in the final Poisson regression model were used. All statistical analyses were performed using Stata (version 12.0) (Stata Corp., College Station, TX, USA) at a 5% significance level.

### Ethical considerations

The “Health and Nutrition Day” study protocol was approved by the Research Ethics Committee of the National School of Public Health at Fundação Oswaldo Cruz. The purposes of the study were explained to all parents/caregivers, and data were only collected after they had signed an informed consent form agreeing to their participation.

## RESULTS

A total of 2,209 children (49.3% males) were studied. The prevalence of stunting was 13.1% while the prevalence of overweight was 7.8%. Most families (85.8%) had a lower socioeconomic status (Class D and E), and more than half (51.4%) of the heads of household had low schooling.

Among the 2,209 children studied, 38.1% were on exc-BF for ≥6 months. Even after adjusting for potential confounding factors, the risk of overweight was lower among these children than those who were exc-BF for <6 months (PR=0.63, 95% CI 0.45-0.89).

Mean weight-for-length z-scores were also lower in children who were exc-BF for ≥6 months (0.367±0.04 vs 0.507±0.03; p=0.003), with a mean difference of 0.140 (95% CI 0.05-0.23).

The multiple regression analysis showed that exc-BF for ≥6 months yielded 0.13 lower (95% CI 0.23-0.03) weight-for-length z-scores.

In addition to exc-BF for ≥6 months, socioeconomic status and birthweight were also independently associated with overweight (Table). There was a lower proportion of overweight in children from families with the lowest socioeconomic status (Class E) than those from the other classes. There was also a positive association with birthweight, i.e. the higher the birthweight, the greater the likelihood of being overweight at age 12-24 months.

## DISCUSSION

The present study found that there is a protective effect of breastfeeding against overweight as early as the second year of life. However, this association was only evidenced following at least six months of exc-BF; von Kries *et al*. (24) found that longer breastfeeding was inversely correlated with overweight and showed that this benefit is evidenced not only in young children but also in school children and adolescents.

Two meta-analyses have reported protective effects of breastfeeding. Arenz *et al*. (25) assessed nine studies with more than 69,000 participants and found that breastfeeding seems to have a small but consistent protective effect against overweight in children. Owen *et al*. (26) analyzed 29 studies, 28 of which showed that breastfeeding was a protective factor against overweight.

In light of this evidence, it is important to explore the biological plausibility of this protective effect of breastfeeding, i.e. potential mechanisms that might interfere with the child's body composition. There is some evidence that it might be related to the adverse metabolic consequences of excessive protein intake (27–29).

Some authors have argued that bioactive compounds, such as leptin, adiponectin, ghrelin, resistin, and obestatin may also play a role in the physiological action of human milk (30–32). Others link the protection to adipokines in human milk, which are responsible for the regulation of various metabolic pathways and play a role in food intake control, nutrient utilization, and potential neuroendocrine modulation of body-weight control. It is, therefore, possible that they contribute to the protective effect of human milk against overweight (32,33).

**Table.** Prevalence of overweight, crude and adjusted prevalence ratios in children aged 12 to 24 months according to the duration of exclusive breastfeeding and socioeconomic, demographic and health-related variables. Semi-arid region, Brazil, 2005

Variable	Category	Frequency No. (%)	Overweight prevalence (%)	Crude PR (95% CI) p value	Adjusted PR^a^ (95% CI) p value
Duration of exclusive breastfeeding, in months (n=2,209)	≥6	841 (38.1)	5.71	0.62 (0.45-0.86) 0.004*	0.63 (0.45-0.89)*
	<6	1,368 (61.9)	9.14	1	1
Socioeconomic classification (n=1,925)	A+B+C	274 (14.2)	12.41	1	1
	D	793 (41.2)	7.94	0.64 (0.43-0.95) 0.027*	0.69 (0.46-1.03)
	E	858 (44.6)	6.06	0.49 (0.32-0.74) 0.001*	0.52 (0.33-0.80)*
Level of education of head of household (n=2,044)	≥5 years	994 (48.6)	8.55	1	NA
	≤4 years	1,050 (51.4)	7.05	0.82 (0.61-1.11) 0.205	
Gender (n=2,209)	Male	1,090 (49.3)	8.35	1	NA
	Female	1,119 (50.7)	7.33	0.88 (0.66-1.17) 0.37	
Area (n=2,163)	Urban	1,654 (76.5)	8.28	1	NA
	Rural	509 (23.5)	6.68	0.81 (0.56-1.16) 0.244	
Skin colour/race (n=2,205)	White	531 (24.1)	9.23	1	1
	Non-white	1,674 (75.9)	7.35	0.80 (0.58-1.09) 0.158	1.01 (0.71-1.45)
Age in months (n=2,209)	12 to 18.0	1,237 (56.0)	8.08	1	1
	18.1 to 24	972 (44.0)	7.51	0.96 (0.93-1.01) 0.173‡	0.96 (0.92-1.01)‡
Height-for-age^a^ (n=2,209)	Normal (z ≥−2)	1,919 (86.9)	7.66	1	1
	Stunting (z <−2)	290 (13.1)	8.97	1.11 (0.98-1.25) 0.088‡	0.97 (0.84-1.12)‡
Birthweight in kg (n=2,164)	Low (<2.5)	157 (7.3)	3.2	1	1
	Underweight (2.5-2.9)	461 (21.3)	4.3	1.36 (0.52-3.56) 0.52	1.59 (0.54-4.68)
	Normal (3.0-3.9)	1,389 (64.2)	8.1	2.53 (1.05-6.11) 0.039*	3.02 (1.11-8.23)*
	High (≥4.0)	157 (7.2)	21.0	6.60 (2.64-16.5) <0.001*	8.98 (3.11-25.9)*
Prenatal care (n=2,196)	Yes	2,131 (97.0)	7.74	1	1
	No	65 (3.0)	12.31	1.59 (0.82-3.09) 0.17	1.89 (0.89-4.02)
Diarrhoea in the last 15 days (n=2,197)	No	1,663 (75.7)	8.24	1	NA
	Yes	534 (24.3)	6.55	0.80 (0.56-1.14) 0.211	

*Statistically significant difference (p≤0.05)

‡Analyzed as a continuous variable

^a^The following variables were included in the multivariable analysis: duration of exclusive breastfeeding; socioeconomic classification, skin colour, age, height-for-age, birthweight, and prenatal care

PR=Prevalence ratio estimated by Poisson regression with robust adjustment of variance, 95% CI=95% confidence interval

NA=Adjusted analysis was not performed as p<0.2 was not achieved in the crude analysis

Exposure to breastmilk components during the first months of life is the earliest nutritional experience of the newborn and is related to metabolic imprinting (34), a term that refers to the effect of nutrition in early life when nutrient intake has lasting effects on the regulation of energy balance and plays an important role in the programming of body-weight (35).

In the assessment of the relationship between breastfeeding and overweight, it should be questioned whether the lower prevalence of overweight among breastfed children is due to breastmilk-related factors or to the fact that children who are not breastfed become more exposed to a diet of high energy density and proteins. In fact, in northeastern Brazil, where most of the Brazilian semi-arid region is located, the prevailing cultural practice is to introduce other foods at a very early age, such as food preparations based on cow's milk with a thickened mixture of complex carbohydrates sweetened with sucrose (36). Unfortunately, there were no data available on children's feeding, which is a limitation of this study.

National Breastfeeding Programme of the Brazilian Ministry of Health was launched in 1981 since when it has been implemented throughout the country, including the semi-arid region. With the support of all state and municipal departments of health and various civil society organizations, it has contributed greatly to the adoption of breastfeeding in Brazil. Its main actions include mass campaigns, training of health workers, individual and group counselling and health education, production and distribution of educational materials, establishment of support groups in the community, and approval of regulations to protect breastfeeding and for the marketing of formula milk (37). The WHO recommends exclusive breastfeeding for the first six months of life (38). This study found that 61.9% of the children did not meet this recommendation, and that 9.5% were not breastfed at all, which is quite a major concern given the social and biological vulnerability of these children. These data suggest that public policy managers should make an even greater effort to improve the effectiveness of the programme.

As mentioned before, overweight children are more likely to become obese adults (1,2). Thus, overweight should be prevented as early as possible. Although every effort should be made to prevent a widespread increase in obesity, including interventions in the pre-pregnancy period in women of childbearing age, the first two years of life represent a unique opportunity to establish healthy eating and behavioural habits that remain throughout an individual's life (39).

### Conclusions

It can be concluded that exclusive breastfeeding for the first six months of life is a protective factor against overweight in children in the second year of life. More effective actions need to be taken to promote breastfeeding among mothers, especially for those living in areas of high social exclusion, such as the Brazilian semi-arid region.

**Conflict of interest:** Authors declare no competing interests.
